# Small Molecules Which Improve Pathogenesis of Myotonic Dystrophy Type 1

**DOI:** 10.3389/fneur.2018.00349

**Published:** 2018-05-18

**Authors:** Marta López-Morató, John David Brook, Marzena Wojciechowska

**Affiliations:** ^1^Queen’s Medical Centre, School of Life Sciences, University of Nottingham, Nottingham, United Kingdom; ^2^Polish Academy of Sciences, Department of Molecular Genetics, Institute of Bioorganic Chemistry, Poznan, Poland

**Keywords:** myotonic dystrophy type 1, myotonic dystrophy type 1 pathogenesis, sequestration of *muscleblind*-like 1, antisense oligonucleotides, aberrant alternative splicing, small molecule compounds

## Abstract

Myotonic dystrophy type 1 (DM1) is the most common muscular dystrophy in adults for which there is currently no treatment. The pathogenesis of this autosomal dominant disorder is associated with the expansion of CTG repeats in the 3′-UTR of the *DMPK* gene. *DMPK* transcripts with expanded CUG repeats (CUG^exp^*DMPK*) are retained in the nucleus forming multiple discrete foci, and their presence triggers a cascade of toxic events. Thus far, most research emphasis has been on interactions of CUG^exp^*DMPK* with the *muscleblind*-like (MBNL) family of splicing factors. These proteins are sequestered by the expanded CUG repeats of *DMPK* RNA leading to their functional depletion. As a consequence, abnormalities in many pathways of RNA metabolism, including alternative splicing, are detected in DM1. To date, *in vitro* and *in vivo* efforts to develop therapeutic strategies for DM1 have mostly been focused on targeting CUG^exp^*DMPK via* reducing their expression and/or preventing interactions with MBNL1. Antisense oligonucleotides targeted to the CUG repeats in the *DMPK* transcripts are of particular interest due to their potential capacity to discriminate between mutant and normal transcripts. However, a growing number of reports describe alternative strategies using small molecule chemicals acting independently of a direct interaction with CUG^exp^*DMPK*. In this review, we summarize current knowledge about these chemicals and we describe the beneficial effects they caused in different DM1 experimental models. We also present potential mechanisms of action of these compounds and pathways they affect which could be considered for future therapeutic interventions in DM1.

## Introduction

Myotonic dystrophy type 1 (DM1) is the most common muscular dystrophy in adults leading to disability and shortened lifespan (1). There is currently no treatment for DM1. The symptoms of this disease include myotonia, muscle weakness and wasting, cardiac conduction defects, diabetes and insulin resistance, and cataracts. DM1 belongs to a larger group of microsatellite disorders associated with expansions of simple repetitive elements within specific genes (2). This autosomal dominant disease is caused by the expansion of CTG repeats in the 3′-UTR of the *DMPK* gene and its pathogenesis is mediated, at least in part, by a toxic RNA *gain-of-function* mechanism. Molecular hallmarks of DM1 cells expressing mutant *DMPK* transcripts (CUG^exp^*DMPK*) are nuclear RNA foci. Their presence has an adverse effect on host cells leading to a broad spectrum of abnormalities. Recent studies of the dynamics of CUG repeat foci have revealed that these are unstable, constantly aggregating, and disaggregating structures (3), and *muscleblind*-like (MBNL)1 protein is directly involved in the stochastic process of foci formation. Being associated with CUG^exp^*DMPK*, the MBNL1 protein has a role in stabilizing RNA aggregates and its downregulation resulted in the decrease of RNA foci accumulation (3). However, MBNL1 depletion does not completely eliminate CUG foci, which suggests involvement of other proteins in mutant transcripts retention. In fact, as demonstrated experimentally, proteins other than MBNL1 may be recruited to the CUG repeat inclusions. Such recruitment may involve limited colocalization, as shown for hnRNP H, hnRNP F, and DDX6 proteins, or it may represent only close association with the RNA foci as shown for SC35 protein (4–7). In addition to the depletion of proteins in CUG RNA foci, DM1 pathogenesis also involves aberrant protein synthesis and/or their altered stability as shown for CUGBP1 protein (8, 9).

*Muscleblind*-like 1 and CUGBP1 are antagonistic regulators of splicing. MBNL1 is a zinc finger protein which recognizes both RNA sequence (YGCY) and structural elements (hairpins) containing pyrimidine mismatches on either normal splicing substrates or pathogenic mutant repeat RNA (10). As shown *in vitro*, the protein binds selectively to the stem region of expanded CUG RNA, and such interaction is detected in DM1 cells as MBNL1 sequestration and colocalization with mutant CUG repeats (11, 12). On the other hand, CUGBP1 protein binds *in vitro* to single-stranded regions of GU-containing transcripts; however, the protein is not enriched in the RNA foci. In DM1 cells, CUGBP1 becomes hyperphosphorylated, stabilized, and consequently, overexpressed (9). Changes in cellular levels and activities of MBNL1 and CUGBP1 proteins result in the abnormal expression of embryonic splice variants in adult tissues which is one of the molecular hallmarks of DM1 pathogenesis. Besides regulating splicing, both proteins are also involved in mRNA translation, RNA stability, protein secretion, and localization of alternative 3′UTR isoforms (13, 14). Their altered activity in DM1 cells also correlate with changes in signaling pathways of various protein kinases including cyclin-dependent kinases (CDKs), glycogen synthase kinase 3β (GSK3β), AKT, and protein kinase C (PKC) (8, 15, 16).

*In vitro* and *in vivo* efforts to develop DM1 therapeutic strategies have been mostly aimed at destroying the toxic ribonucleoprotein complexes *via* targeting the mutant CUG^exp^RNA and/or inhibiting its pathogenic interactions with MBNL1 protein, leading to the generation of several strategies that proved to have a beneficial effect in DM models (17, 18). Thus far, antisense technology that utilizes synthetic siRNAs (19), modified CAG antisense oligonucleotides (20–22), viral vector-mediated expression of hU7-snRNA-(CAG) (23), or a hammerhead RNA (ribozyme) (24) designed to cut CUG repeats, appear effective in DM1 cells and mouse models of the disease. Moreover, morpholino CAG oligonucleotides (25, 26) and several bioactive small molecules (27–34), which are CUG repeat binders, have been reported as potential therapeutic agents for DM1, capable of inhibiting the interactions between expanded CUG RNA and MBNL1 protein.

As our understanding of the pathogenesis of DM1 has grown over the past years, the focus of the research encompassed more molecular events being important for the progression of the disease, e.g., aberrantly spliced genes, RAN translation (35), and deregulation of miRNAs (36). Considering that DM1 is a multisystem disorder, it seems reasonable to target multiple molecules and pathways of the misregulated DM1 apparatus with the aim of developing a beneficial strategy to combat DM1.

Over the past few years, experimental evidence has indicated that, indeed, small molecule chemicals affecting different cellular pathways independently of CUG^exp^*DMPK* can mitigate some of the molecular hallmarks of DM1 pathogenesis (18). However, it remains elusive, how the molecules alleviate DM1 features. Their efficiency, though, indicates that it is reasonable to search for novel candidate therapeutic targets that will provide new opportunities for future studies aiming to decipher the complex pathomechanism of DM1. In this review, we summarize current knowledge about such molecules, acting independently of direct binding to CUG^exp^*DMPK*, and we describe beneficial effects they caused in different DM1 experimental models. We also present potential mechanisms of action of these compounds and cellular pathways they affect which could be considered for future therapeutic interventions in DM1.

## Inhibitors of Transcription

**Figure 1:**
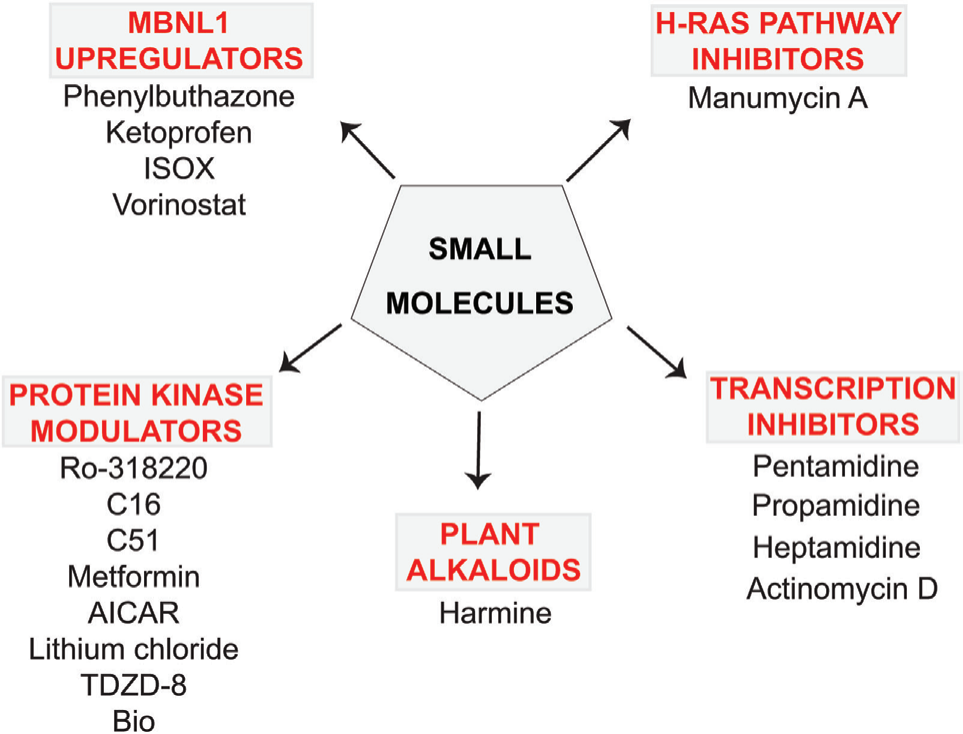
Small molecule compounds alleviating myotonic dystrophy type 1 (DM1) pathogenesis. Small molecule compounds which mitigated DM1 pathogenesis in different experimental systems are shown. The molecules are classified into a few categories depending on their presumable mechanisms of action. All the molecules are believed to act independently of direct interactions with expanded CUG repeats RNA.

A few compounds that inhibit transcription and alleviate some of the molecular symptoms of DM1 have been identified in recent years. These include pentamidine and its analogs, as well as actinomycin D (ActD) (Figure 1) (37–40).

### Pentamidine

Pentamidine is an FDA-approved drug currently used to treat patients with *Pneumocystis carinii* infections (pneumonia) in acquired immunodeficiency syndrome, as well as patients with *Trypanosomiasis* and *Leishmaniasis* infections (41, 42). Pentamidine is a diamide composed of two phenyl amidine groups joined by a five-carbon methylene linker (Table 1). It has been speculated to inhibit the splicing of essential group I introns in *P. carinii*, preventing its growth (43, 44), and it was recently shown to inhibit translation by binding tRNA (45). Pentamidine may also block DNA replication (41), since its structure with DNA showed binding in the minor groove of the double-stranded molecule (46). Therefore, it is likely to interact with many different nucleic acid targets within cells. Interestingly, in DM1 patient’s cells and model organisms’ pentamidine disrupted CUG RNA foci, released MBNL1 protein, and reversed aberrant alterative splicing of some pre-mRNAs typically misspliced in DM1. Initially, these effects were thought to be caused by binding of the compound to the CUG repeats, because of its ability to bind DNA (40), however, subsequently it was found that its effect on the DM1 molecular phenotype was attributed to either decreased transcription of the CUG transcripts or increased degradation of the toxic RNA (38). So far, the beneficial effects of pentamidine have been shown in HeLa and HEK293 cells transfected with 960 CUG repeats and in model organisms, including the HSA^LR^ mice and a *Drosophila* model expressing 250 CTG repeats (37, 40) (Table S1 in Supplementary Material). Treatment of HeLa cells transiently expressing CUG repeats and transgenic HSA^LR^ mice showed, respectively, a decrease of CUG mRNA expression (and subsequent reduction of CUG RNA foci number), and a reduction of the HSA transcript. Correction of some misspliced pre-mRNAs, i.e., *cTNT* E5 and *INSR* E11 (in HeLa cells) and *Clcn1* E7a and *Serca1* E22 (in HSA mice) was attributed to the liberation of MBNL1 protein from diminished CUG foci and reduction of CUG transcript levels. This suggests that pentamidine does not directly block MBNL1 binding to the repeats and supports the hypothesis that it either inhibits transcription of the CTG repeats or increases the rate of CUG^exp^RNA degradation. However, in a DM1 *Drosophila* model, a behavioral and molecular improvement which included a minor rescue of cardiac defects reduced CUG foci, and Mbnl1 displacement was not correlated with diminished expression level of CUG repeats mRNA (37) (Table S1 in Supplementary Material). It was suggested that the beneficial effects induced by pentamidine are due to Mbnl1 diffusion and subsequent dispersion of toxic RNA in the nucleus, rather than by inhibition of transcription of the toxic RNA or its degradation.

**Table 1:** Molecular formula and chemical structure of small molecule compounds which do not directly target expanded CUG repeats and alleviate pathogenesis of myotonic dystrophy type 1.

Name	Molecular formula	Structure	Potential mechanism of action
Pentamidine	C19H24N4O2	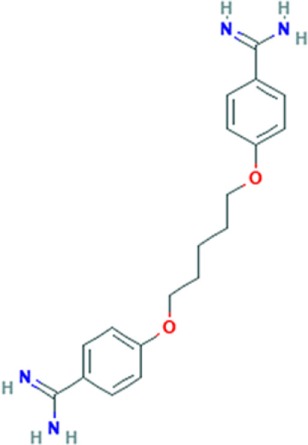	Inhibition of transcription
	
Propamidine	C17H20N4O2	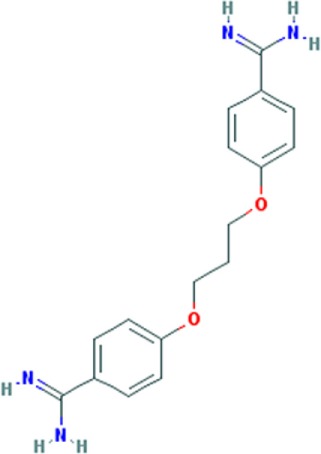	
	
Heptamidine	C21H28N4O2	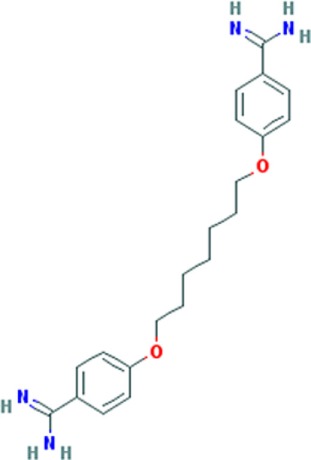	
	
Compound 13	C18H16N4O	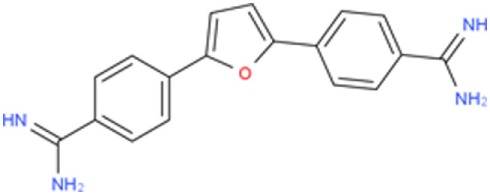	
	
Actinomycin D	C62H86N12O16	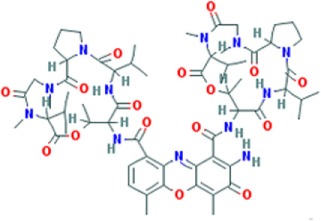	

Phenylbuthazone	C19H20N2O2	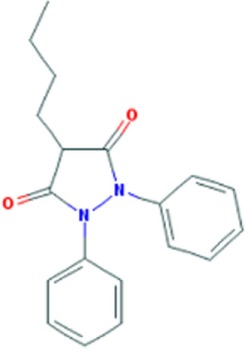	Upregultion of *muscleblind*-like 1
	
Ketoprofen	C16H14O3	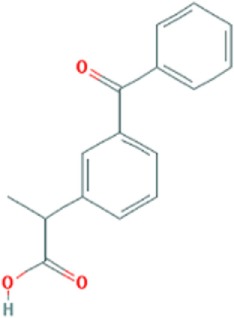	
	
ISOX	C22H30N4O6	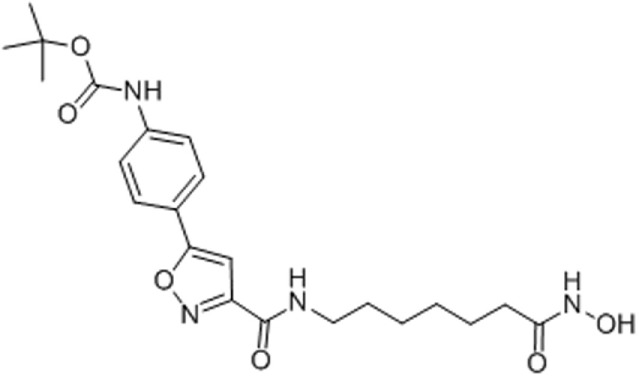	
	
Vorinostat	C14H20N2O3	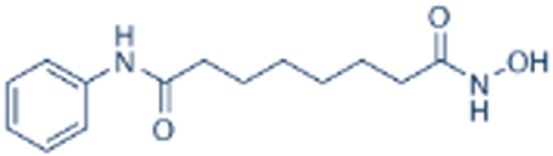	

Manumycin A	C31H38N2O7	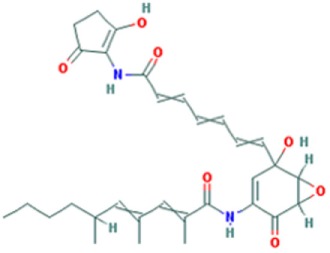	Inhibition of H-RAS pathway

Metformin	C4H11N5	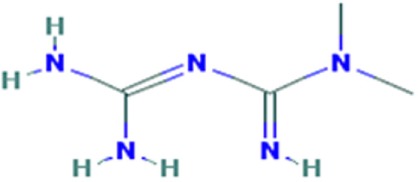	Modulation of protein kinases
	
AICAR	C9H14N4O5	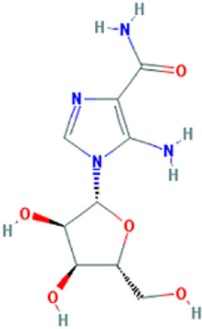	
	
Ro 31-8220	C25H23N5O2S	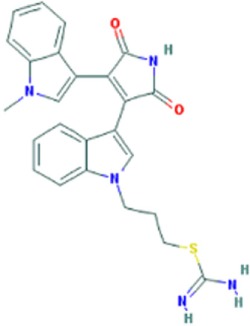	
	
C16	C13H8N4OS	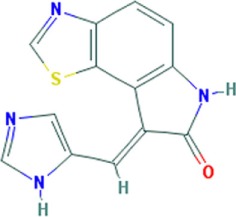	
	
C51	C23H21N5	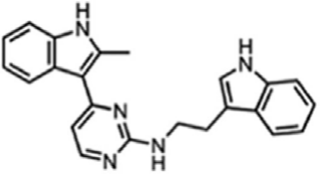	
	
Lithium chloride	LiCl	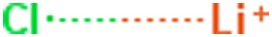	
	
TDZD-8	C10H10N2O2S	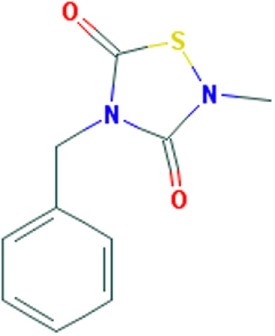	
	
6-Bromoindirubin-3′-oxime	C16H10BrN3O2	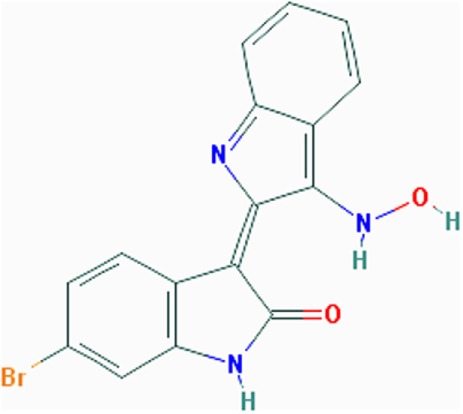	

Harmine	C13H12N2O	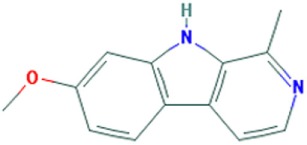	Undetermined

### Pentamidine Analogs

The results obtained with pentamidine as an anti-DM1 drug were promising; however, they showed a certain degree of toxicity and thus needed optimization, and the mechanism of action (MoA) needed clarification. To improve the physiochemical properties of pentamidine and to decrease its toxicity, a series of analogs of pentamidine, containing between three to nine methylene carbons, were analyzed for their ability to rescue splicing of transiently transfected minigenes in a HeLa cell model of DM1 and in HSA^LR^ mouse model (Table S1 in Supplementary Material). Two different minigenes, i.e., *cTNT* E5 and *INSR* E11, containing exons that are misspliced in DM1 were tested. Propamidine (methylene linker length of 3) and heptamidine (methylene linker length of 7) (Table 1) were identified as the most promising analogs in rescuing missplicing in both DM1 models (38). Although analogs with more methylenes rescued missplicing more efficiently, an increase of the methylene linker length also reduced solubility and increased toxicity. Interestingly, in the HeLa cell model only propamidine was able to reduce CUG repeat transcript levels in a dose-dependent manner (however, not as effectively as pentamidine), whereas no reduction was observed with heptamidine treatment before significant cell death occurred. The *cTNT* splicing was improved to varying degrees with different linker analogs, however, no correction was observed with propamidine at concentrations which were not toxic. Furthermore, the improvement was not dependent on inhibition of CUG transcript expression. Unlike the *cTNT*, all linker analogs partially or fully rescued the missplicing of *INSR* E11 after CUG repeats expression. Different MBNL targets may require different concentrations of the proteins to be properly regulated, which could explain the results found regarding correction of alternative splicing of different genes (47).

Treatment of HSA^LR^ DM1 mice with heptamidine caused reduction of both the mRNA and pre-mRNA levels of the transgene, while in HSA^SR^ mice expressing short CTG repeats, no such reduction was detected. This suggests that the effect was dependent on the presence of extended repeats, rather than the HSA promoter, transgene, or the short repeats. In the HSA^LR^ mouse model, heptamidine completely reversed the missplicing of *Clcn1* E7a, while splicing of *Atp2a1* E22 was rescued partially and at lower concentrations than pentamidine. In addition, heptamidine caused a rapid (after 1 week) and significant reduction of myotonia, as well as diminished levels of HSA^LR^ transcript (Table S1 in Supplementary Material). Nevertheless, it showed high toxicity in the mouse model. However, variation of the linker length of pentamidine resulted in significant improvements and is encouraging to develop a therapy for DM1. Reduction of the CUG transcript in *in vitro* transcription assays as well as in both DM1 models, i.e., HeLa cells and HSA^LR^ mice, as well as reduced formation of nuclear foci, suggest that pentamidine and its analogs most likely inhibit transcription of CTG*CAG repeat DNA. However, other possible mechanisms cannot be ruled out as pentamidine is a DNA-binding molecule and may affect expression of other genes, some of which may be regulators of alternative splicing.

Further modifications of pentamidine and heptamidine to modify their size, solubility, degrees of freedom, number of hydrogen bond donors, and hydrophobicity led to the generation of new promising compounds. These molecules were tested for their ability to correct splicing defects in cellular and transgenic mouse models of DM1, as well as their toxicity relative to the parental compounds (48). Three different series of molecules varying in amidine substitution, central linker length, and planarity were synthesized. Compound 8, a molecule with an un-substituted propyl chain, was able to rescue missplicing in the DM1 HeLa cell model with no toxicity at the range of concentrations used, but it was half as effective as pentamidine. Modification of the planarity produced a linear molecule, compound 12, and a molecule with a concave shape, compound 13 (Table 1). Both compounds rescued missplicing similarly to pentamidine and they were not toxic over the range of concentrations needed for the rescue, unlike pentamidine. Compound 12 affected splicing in a DM-independent manner in a model without CUG expanded repeats, suggesting a modulation of splicing through a mechanism beyond targeting the CUG repeats. On the contrary, compound 13 only rescued alternative splicing in the presence of CUG repeats. Furthermore, it reduced foci in the DM1 HeLa cell model and partially rescued misplicing of *Clcn1* E7a and *Atp2a1* E22 in the HSA^LR^ mouse model at a similar level observed with heptamidine, but without the associated toxicity (39).

The MoA of pentamidine has been previously proposed to be inhibition of transcription through binding to the CTG repeats or reduction of the stability of the transcript. Nonetheless, binding to the CUG repeats and therefore displacement of MBNL1 proteins, especially in the case of the analogs generated has not been completely ruled out, and further work is required to clarify the MoA of the diamines as anti-DM1 drugs.

### Actinomycin D

Actinomycin D (Table 1) is a polypeptide antibiotic which forms a stable complex with double-stranded DNA, inhibiting DNA-primed RNA synthesis and causing single-stranded breaks in DNA, therefore stopping the proliferation of cells (49, 50). ActD is an FDA-approved anti-cancer drug with activity to inhibit global transcription. More recently, this antibiotic was shown to improve the DM1 molecular phenotype (39). ActD does not bind to CUG repeat RNA *in vitro*, however, low concentrations, insufficient to affect global transcription, triggered a significant reduction of expression of the expanded CUG repeat RNA in HeLa cells and in DM1 fibroblasts, by 50–70% and by 44–60%, respectively. At effective dosages, ActD was mildly toxic to HeLa cells causing reduction of nuclear CUG^exp^RNA foci by 50% and releasing MBNL1 from the foci. In HSA^LR^ mice, the molecule specifically reduced mRNA levels of the repeat-containing HSA transgene and completely rescued the aberrant splicing of *Clcn1* E7a, whereas partially corrected splicing of a few other genes, i.e., *Atp2a1* E22, *Mbnl1* E7, *Vps39* E3, *Nfix* E7, and *Ldb3* E11. Importantly, at the range of dose used these changes were not correlated with global inhibition of transcription (Table S1 in Supplementary Material). The MoA of ActD in DM1 is yet to be determined. However, its effects produced in the disease models suggest inhibition of transcription of the CUG^exp^RNA by binding to the CTG repeats in DNA and blocking the RNA polymerase. Such mechanism is feasible since it is known that ActD intercalates into GC-rich sequences, stabilizing topoisomerase-I DNA complexes and preventing RNA polymerase progression (51).

## Compounds Upregulating MBNL1

Overexpression of MBNL1 protein has been shown to alleviate pathogenesis of DM1 by reversal of aberrant alternative splicing and rescue of myotonia (52). Accordingly, small molecules which upregulate expression of the splicing factor would have a potential of becoming therapeutic molecules for DM1. Two compounds with such capacity have been identified by Chen et al. (53). Phenylbutazone (PBZ) and ketoprofen (Table 1) belong to nonsteroidal anti-inflammatory drugs (NSAIDs) which are used to reduce pain and fever, prevent blood clots, and in higher doses, decrease inflammation. NSAIDs inhibit the activity of cyclooxygenase enzymes (COX-1 and/or COX-2). These enzymes participate in the synthesis of key biological mediators in the cells, such as prostaglandins which are involved in inflammation, and thromboxanes which are involved in blood clotting (54, 55).

It was shown that PBZ enhances MBNL1 expression in proliferating and differentiating C2C12 cells in a dose-dependent manner (53) (Table S1 in Supplementary Material). Consistent with this result, when analyzed in HSA^LR^ DM1 mice PBZ elevated the expression of *Mbnl1 mRNA* and protein in tibialis anterior and in quadriceps muscles. Interestingly, the expression of CUGBP1 remained unchanged after the treatment with PBZ. Consequently, the treated mice showed partial rescue of aberrant splicing of MBNL1-dependent exons, such as *Clcn1* E7a, *Nfix* E7, and *Rpn2* E17, whereas no splicing correction was found for CUGBP1-regulated exons. Amelioration of the molecular features in HSA^LR^ mice was further confirmed in behavioral and histological tests and the mice had an increase of grip strength and decrease in the number of muscle fibers with central nuclei (53). Although PBZ elevated Mbnl1 expression levels in HSA mouse muscles, colocalization of the protein with CUG RNA foci was markedly attenuated by its treatment. This result suggests that PBZ inhibits the interaction between CUG RNA foci and MBNL1 and reduces the ratio of MBNL1 in the mutant transcript. The precise MoA of PBZ in DM1 remains unclear and it is speculated that it may not be limited to the liberation of MBNL1 proteins from CUG^exp^RNA. The observed upregulation of *MBNL1* mRNA was attributed to demethylation of its intron 1, making this region to act as an enhancer of transcription (53).

Ketoprofen is another NSAID which was able to upregulate MBNL1 levels in C2C12 cells (53). In a *Drosophila* model of DM1 expressing 480 interrupted CUG repeats the compound suppressed CUG-mediated lethality (56). The MoA of ketoprofen in DM1 has not yet been elucidated.

More recently, a flow cytometry-based screen led to identification of small compounds that upregulated MBNL1 and partially rescued splicing defects in DM1 patient-derived fibroblasts (57). Using engineered HeLa cells containing a ZsGreen fluorescent tag in the N-terminus of the MBNL1 sequence, the HDAC inhibitors ISOX, and Vorinostat (Table 1) were found to produce a 2- and 1.8-fold increase of the ZsGreen-MBNL1 signal, respectively (57). Treatment of either normal or DM1 fibroblasts with ISOX and Vorinostat produced a significant increase of MBNL1 levels, and partially rescued the splicing of *SERCA1* e22 and *INSR* e11 in both cell lines (Table S1 in Supplementary Material). Since ISOX and Vorinostat are HDAC inhibitors, they may affect expression of *SERCA1, INSR*, and *DMPK* mRNAs. However, as shown by Zhang and coauthors, treatment of normal and DM1 fibroblasts with these molecules caused no significant changes in the genes levels. The MoA of ISOX and Vorinostat is not fully clear, but inhibition of HDAC appears to have a role in modulating MBNL1 levels, and the effects might be caused by inhibition of several HDACs. ISOX inhibits HDAC6 at low concentrations, but also inhibits HDAC1 and other HDACs at higher concentrations. Vorinostat is an FDA-approved HDAC inhibitor for the treatment of cutaneous T cell lymphoma, and inhibits class I and class II HDACs, altering gene transcription and causing cell cycle arrest. Nonetheless, in the light of these recent results, modulation of MBNL1 through mechanisms other than inhibition of HDAC cannot be completely ruled out (57).

## Inhibitors of H-Ras Pathway

**Figure 2:**
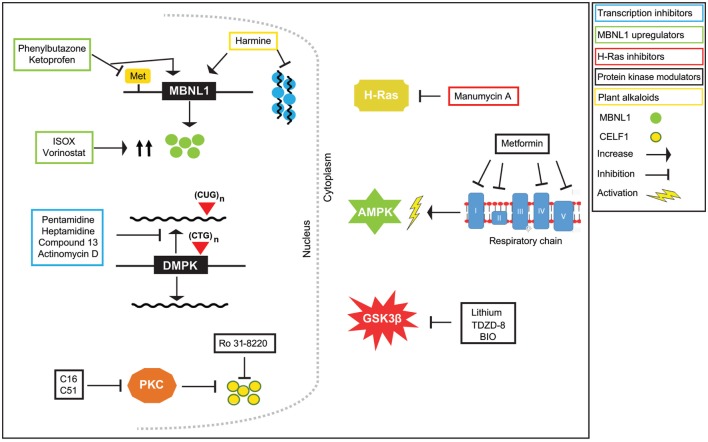
Therapeutic effects of small molecules on myotonic dystrophy type 1 (DM1) pathogenesis. DM1 is characterized by the presence of RNA foci which are aggregations of the mutant CUG^exp^ transcript with *muscleblind*-like (MBNL)1 and other proteins. CUGBP1 is not sequestered by foci, but it is upregulated. The imbalance of these two alternative splicing proteins causes the aberrant alternative splicing of many pre-mRNAs. Treatment of DM1 cells and model organisms with small molecules that target the DNA and/or affect proteins involved in the DM1 pathogenesis can lead to beneficial effects, such as inhibition of transcription of the mutant transcript, or its degradation, release of MBNL1 protein from RNA foci, downregulation of CUGBP1 protein, and ultimately the correction of the aberrant splicing.

The Ras family includes three members: H-Ras, K-Ras, and N-Ras. They play roles in a large number of biological processes including cell morphology, survival, apoptosis, gene expression, and alternative splicing regulation (58). Posttranslational modifications of Ras proteins lead to their activation and these modifications include farnesylation of H-Ras, as well as farnesylation and geranylgeranylation of K-Ras and N-Ras (59). Manumycin A (Table 1) is an FDA-approved antibiotic that acts as a potent and selective farnesyltransferase (FTase) inhibitor (60). By inhibiting FTase, manumycin A prevents activation of H-Ras protein but has no effect on K-Ras and N-Ras. It has been reported that some of DM1 features can be alleviated by exposure to manumycin A (Figure 2) (61). Treatment of HSA^LR^ DM1 mice with the compound led to correction of *Clcn1* E7a splicing, what was linked to the inhibition of H-Ras activity since siRNA knockdown of endogenous H-Ras protein recapitulated improvement of the splicing. Such effect was not detected when the two other Ras proteins were downregulated. Although skeletal muscle injections with manumycin A corrected aberrant splicing of *Clcn1* in DM1 mice, splicing of two other genes, *Serca1* E22 and *m-Titin* Mex5, was not altered (61). Importantly, in this experimental model of DM1, manumycin A did not alter expression of Mbnl1 and Cugbp1, which are involved in splicing regulation of *Clcn1* E7a, *Serca1* E22, and *m-Titin* Mex5 (Table S1 in Supplementary Material). Thus, it was concluded that the effect of manumycin A on aberrant splicing was independent of these two splicing factors. Therefore, it is possible that manumycin A, which acts as a Ras FTase inhibitor, triggers alterations in H-Ras signaling. This may influence a *trans-*acting factor(s) other than MBNL1 and CUGBP1 involved in alternative splicing and may contribute to chloride channel splicing. However, a category of *trans-*acting factor(s) affected remains unknown.

## Modulators of Protein Kinases

### Protein Kinase Inhibitors

Pathogenesis of DM1 has been linked to disrupted protein kinase signaling pathways. Altered expression and nonspecific activation have been shown for PKC, Src family kinases, CDKs, GSK3β, and protein kinase AKT (8, 15, 16). Current studies in DM1 with the use of small molecule inhibitors of protein kinases have shed light on a potential alternative strategy in DM1 therapeutic intervention. Screening of several libraries of small molecule compounds, including phosphatase and kinase inhibitors, using a medium throughput phenotypic assay by Ketley et al. led to the identification of such molecules (62). Based on the identification of nuclear foci in DM1 cells using *in situ* hybridization and high-content imaging, Ro 31-8220 (Table 1) was identified as a compound of potential therapeutic benefit in DM1. The compound eliminated nuclear CUG^exp^RNA foci, reduced MBNL1 protein in the nucleus, affected *SERCA1* E22 alternative splicing, and decreased steady-state levels of CUGBP1 protein (Figure 2). Ro 31-8220 was previously identified as a PKC inhibitor and seen to affect the hyperphosphorylation of CUGBP1 and ameliorate the cardiac phenotype in a DM1 mouse model (8, 63). Nonetheless, Ketley et al. demonstrated that Ro 31-8220 acts independently of PKC on DM1 pathomechanism, suggesting the involvement of other kinases. Although the MoA of Ro-31-8220 still requires further investigation, the compound is likely to work independently of CUG repeat RNA binding.

Another study has identified two compounds, C16, an imidazolo-oxindole inhibitor and C51, a pyrimidine-based inhibitor (Table 1), of kinase inhibitory activity with the potential to alleviate DM1 molecular phenotype (64). Previous studies have described these two compounds as ATP-site directed PKR inhibitors (30, 65); however, activity of C16 against kinases other than PKR has also been reported (66). C16 has been successfully used *in vitro* and *in vivo* as an effective and versatile neuroprotective agent and shown to have potential value in the treatment of neurodegenerative diseases (66–69). Results by Wojciechowska et al. demonstrate that C16 and C51 may have therapeutic potential in patients with DM1 as well. These compounds produced a redistribution of the MBNL1 protein sequestered in CUG^exp^RNA foci and a reduction of the steady-state levels of CUGBP1. These actions were accompanied by correction of the aberrant alternative splicing of MBNL1-dependent (*SERCA1* E22, *DMD* E78, *MBNL1* E7, and *LDB3* E7) and CUGBP1-dependent (*ITGA6* E24, *MTMR3* E16, and *SORBS1* E6) pre-mRNA targets, shifting the patterns of spicing toward those observed in non-DM cells. However, despite causing the nuclear CUG^exp^RNA foci to become less abundant, as determined by *in situ* RNA hybridization and immunocytochemistry for MBNL1 protein, the compounds did not eliminate foci completely.

### GSK3β Inhibitors

Glycogen synthase kinase 3β has been seen to have a role in DM1 pathogenesis (16). This kinase phosphorylates cyclin D3 at T283, which triggers degradation of the cyclin. Cyclin D3 associates with CDK4 and the resulting complex phosphorylates CUGBP1 at S302 and regulates the translational activity of the protein. CUGBP1 regulates translation of proteins important for skeletal muscle development, thus its normal translational activity is required for a proper myogenesis. In muscle biopsies of DM1 patients, GSK3β was found to be increased, causing a degradation of cyclin D3 and a reduced phosphorylation of CUGBP1 at S302, what was linked to delayed myogenesis. The presence of CUG expanded repeats appears to increase the stability of GSK3β by causing the autophosphorylation of the protein at Y216 (16, 70). Altogether, these observations point at GSK3β as a potential therapeutic target in DM1. Indeed, treatment of HSA^LR^ mice with two GSK3β inhibitors, lithium and TDZD-8 (thiadiazolidine) (Table 1), reduced the levels of GSK3β in skeletal muscle and normalized the levels of cyclin D3, restoring CUGBP1 translational function. These changes improved skeletal muscle strength in the mice and reduced myotonia, suggesting that correction of GSK3β may have a beneficial effect on myofiber regeneration (16).

Another GSK3β inhibitor, 6-bromoindirubin-3′-oxime (BIO) (Table 1), was used to treat young HSA^LR^ mice, resulting in recovery of the grip strength to near-normal levels and the effect was maintained for several months after completion of the treatment. In addition, the levels of GSK3β and cyclin D3 in 12-month-old mice previously treated with BIO were similar to those in control mice, and the translational activity of CUGBP1 was corrected. Results from this study suggest that inhibition of GSK3β in young mice is sufficient to maintain corrected levels of the GSK3β-cyclin D3-CUGBP1 pathway and nearly normal muscle health over a long period of time after completion of the treatment (71).

### Activators of AMP-Activated Protein Kinase (AMPK)

Metformin (Table 1) is an FDA-approved antidiabetic drug and was earlier reported to improve hyperglycemia through increased insulin-independent glucose uptake in peripheral muscles of DM1 patients (72). Most recently, metformin was investigated in *in vitro* DM1 models of human embryonic stem cells and in primary myoblasts derived from patients (73). The drug appeared to modify the alternative splicing of a subset of genes associated with DM1 in these cell models, as it partially rescued the aberrant splicing of *INSR* E11, *CLCN1* E7a, *TNNT2* E5, *ATP2A1* E22, and *DMD* E78. The effect on the modification of the alternative splicing has been linked to the inhibition of the complex I of the respiratory chain, which in turn raises the intracellular AMP/ATP ratio, which triggers the activation of AMPK. The role of AMPK activation in alternative RNA splicing was tested in DM1 myogenic progenitor cells and in myoblasts with the AMPK activator AICAR [5-aminoimidazole-4-carboxamide 1-β-d-ribofuranoside, Acadesine, N^1^-(β-d-ribofuranosyl)-5-aminoimidazole-4-carboxamide] (73). Treatment of DM1 cells promoted changes in similar subsets of pre-mRNAs as found for metformin. However, AICAR did not modulate the *INSR* E11 splicing in the cells used. Thus, these results suggest that activation of AMPK is partially involved in alternative splicing modulation, but metformin appears to trigger an additional molecular pathway. Interestingly, metformin decreases tyrosine kinase receptor signaling (73). The tyrosine kinase receptors include the epidermal growth factor receptor, the signaling pathway which controls *INSR* E11 inclusion *via* the inhibition of *hnRNPA1* and *hnRNPA2B1* expression (74, 75). This could explain the effect of metformin on the alternative splicing of *INSR*. The MoA behind other splicing events modulated by metformin but not by AMPK activation remains unclear.

## Small Molecules of Natural Origin

Many small molecules have been synthesized as potential therapies for DM. However, only a few studies have reported utilization of molecules of natural origin (40, 76). Recently, a set of plant-derived alkaloids was identified as small molecules with an anti-DM1 effect (77). Using a novel CUG_78_–MBNL1 complex inhibition assay, a collection of isolated natural compounds and extracts from plants and fungal strains was screened. The bioactivity of the compounds was investigated in human DM1 cells and HSA^LR^ mice resulting in the identification of several alkaloids, including carboline harmine and isoquinoline berberine, which ameliorated certain aspects of the DM1 pathology in these models.

Aromatic alkaloids can interact with RNA, and indeed berberine and harmine (Table 1) have been reported to bind RNA structures (78, 79). In DM1 myoblasts, harmine reduced foci, nevertheless it did not improve the histology in gastrocnemius muscle of the HSA^LR^ mice, as the percentage of fibers with internalized nuclei was not altered by the treatment of the mice with the compound (77). However, although harmine increased the levels of MBNL1 in DM1 myoblasts and enhanced MBNL1-dependent alternative splicing of *cTNT* E5, *INSR* E11, and *Clcn1* E7a (Table S1 in Supplementary Material), a similar effect was found in wild-type myoblasts, suggesting that inhibition of the CUG–MBNL1 complex is not the primary MoA of this alkaloid. Consequently, it was suggested that harmine acts through another mechanism that causes the increase of MBNL1 levels and the amelioration of the spliceopathy. Despite the side effects, harmine represents an interesting small molecule that could be optimized by chemical modifications to become a potential DM1 therapy.

## Conclusion

Myotonic dystrophy type 1 is a life-shortening, debilitating disorder for which there is currently no treatment. Pathogenesis is associated with nuclear retention of mutant DMPK mRNA which attract or is attracted by various proteins. Experimental data suggest that the formation of riboprotein complexes is a necessary trigger for DM1 pathogenesis. Thus, compounds which reduce such inclusions would be therapeutically beneficial. Over the past few years, many efforts have been focused on the synthesis of small molecule chemicals specifically recognizing mutated CUG repeats and either cutting the toxic RNA or blocking their interactions with relevant proteins (19, 22, 26). In both cases, treatment improved DM1 molecular and behavioral features including fewer CUG^exp^RNA foci, liberation of MBNL1 protein, rescue of aberrant alternative splicing, and muscle pathology correction. Interestingly, similar improvements have also been observed with other small molecules affecting the DM1 mutation indirectly (38, 48, 62). Although there are many questions concerning the mode of action of these chemicals, *in vitro* and *in vivo* efficacy underline the notion that these molecules can have therapeutic benefit in DM1. Furthermore, several of the described molecules are FDA-approved drugs, potentially offering an opportunity of repositioning.

The development of therapeutic approaches based on small molecules has several advantages, including lower costs with ease of manufacturing, ease of management of the therapy, with the possibility to rapidly interrupting the treatment in case of toxicity, ease of administration and tissue delivery, opportunities of repositioning, and most notably, that the pharmaceutical industry has decades of experience in refining and improving potentially useful compounds *via* conventional medicinal chemistry-based approaches. Such efforts may prove fruitful for DM1.

## Author Contributions

Conception and design and wrote the main manuscript text: ML-M, MW, and JDB. Prepared tables and figures: ML-M and MW. All authors reviewed the manuscript.

## Conflict of Interest Statement

The authors declare that the research was conducted in the absence of any commercial or financial relationships that could be construed as a potential conflict of interest.
