# Supplementary material for: Small Molecules Which Improve Pathogenesis of Myotonic Dystrophy Type 1
Source: Front Neurol. 2018 May 18;9:349. doi: 10.3389/fneur.2018.00349 (PMC5968088; doi:10.3389/fneur.2018.00349)
Supplement: Supplementary file 1 [file table_1.PDF]

Supplementary Table 1: Small molecules with anti-DM1 activity.

Compound	Model	Active concentration	Biological readout	Potential mechanism of action	Reference
**INHIBITORS OF TRANSCRIPTION**					
**Pentamidine**	HeLa DM1 cell model (960CTG)<br><br>HSA^{LR} mice (ip injection)	25-50µM (for *INSR* correction)<br>50-75µM (for *cTNT*)<br>75µM (for foci, HeLa)<br>50µM (for foci, HEK)<br><br>25mg/kg twice a day<br>40mg/kg once a day	CUG mRNA reduction, foci reduction, MBNL1 displacement, splicing correction of *cTNT* E5 and *INSR* E11<br><br>CUG mRNA reduction, *Clcn1* E7a and *Serca1* E22 improvement	Pentamidine acts *in vivo* by binding CUG repeat RNA and displacing MBNL1, freeing MBNL to regulate alternative splicing.	Warf MB et al, 2009
	(CTG)54 and (CAG)54 *in vitro* transcription assay<br><br>HeLa DM1 cell model (960CTG)<br><br>HSA^{LR} mice	IC50e = 14.2± 4.7 and 13.2 ± 2.3, respectively, for CTG and CAG	Inhibition of transcription *in vitro* (AT-independent manner); inhibition occurs through direct interactions with the DNA itself<br><br>Significant reduction of the levels of CUG transcript	Pentamidine does not bind to CUG repeats and directly block MBNL1 binding as previously proposed, but either decreases transcription of the CUG RNA or increases the rate of degradation.	Coonrod LA et al, 2013
	DM1 *Drosophila*	1µM	Foci reduction, Mbnl1 release and redistributed throughout the nucleus, No CUG mRNA reduction, rescue of heart rhythmicity and contractility	The rescue of the cardiac-dysfunction phenotype achieved by pentamidine was mediated by releasing Mbnl1 sequestration rather than reducing toxic RNA expression level.	Chakraborty M, 2015
**Analogs of pentamidine**<br>Analogues containing 3-9 methylene carbons	HeLa DM1 cell model (960CTG)	80µM	**Propamidine** reduces CUG RNA level (to lesser extent than pentamidine)<br>No reduction with **heptamidine**<br><br>**Hexamidine** and **heptamidine** improved *cTNT* E5 splicing similar to **pentamidine** and **butamidine**<br><br>All linker analogues were able to partially or fully rescue the mis-splicing of *INSR* E11	Pentamidine and analogs are likely to work through binding the CTG*CAG repeat DNA to inhibit transcription.	Coonrod LA et al, 2013

	HSA^{LR} mice	**Heptamidine**<br>20 mg/kg, 7 days<br><br>30 mg/kg, 7 days<br><br>15 mg/kg, 7 days	Splicing rescue of *Clcn1* E7a and *Serca1* E22<br>Decrease of myotonia<br><br>Reduction of the HSA mutated transcript but not the wt HSA transcript		
**Compound 13 (furamidine)**	HeLa DM1 cell model (960 CTG)<br><br>HSA^{LR} mice	80µM<br><br>10-20 mg/kg, 7 days	Rescue of splicing of *cTNT* E5, *INSR* E11<br><br>Foci reduction, diffused nuclear MBNL1, disruption of the CUG:MBNL complex<br><br>Splicing rescue of *Clcn1* E7a and *Serca1* E22	Pentamidine inhibits transcription of CTG DNA or reduce the stability of the transcript.<br><br>Another possible mechanism is that pentamidine binds directly to CUG RNA and therefore displace MBNL proteins from nuclear aggregates.	Siboni RB et al, 2015 (a)
**Actinomycin D**	HeLa DM1 cell model (960 CTG)<br><br>DM1 fibroblasts<br><br>HSA^{LR} mice	10 nM, 18 h<br><br>5–20 nM, 18h<br>1–6 nM, 18 h<br><br>0.025 mg/kg, 5 days<br>0.125–1.25 mg/kg, 5 days	Foci reduction<br><br>Reduced CUG RNA levels<br>Reduced CUG RNA levels<br><br>HSA transcript reduction<br><br>Splicing rescue of *Serca1* E22, *Clcn1* E7a, *Mbnl1* E7, *Vps39* E3, *Nfix* E7 and *Ldb3* E7	Actinomycin D binds to the CTG repeat expansion and blocks the RNA polymerase II, inhibiting transcription of the CUG^{exp} RNA.	Siboni RB et al, 2015 (b)
**COMPOUNDS WHICH UPREGULATE MBNL1 LEVELS**					
**Phenylbutazone**	C2C12 myoblasts<br><br>HSA^{LR} mice	50µM<br><br>16.7mg/Kg/day	Upregulated expression of MBNL1 mRNA up to 1.9-fold in a dose-dependent manner (0.16 µM to 972 µM)<br><br>Upregulated MNBL1 protein level on differentiation day 5<br><br>Significant reduction of colocalization of Mbnl1 and RNA foci caused by disruption of the CUG-Mbnl interaction, *Clcn1* E7a splicing correction and Clcn1 protein upregulation; splicing correction of *Nfix* E7 and *Rpn2* E17, improved	PBZ suppresses methylation of an enhancer region in MBNL1 intron 1, enhancing transcription of *MBNL1* mRNA.<br><br>PBZ attenuates binding of MBNL1 to abnormally expanded CUG repeats *in cellulo* and *in vitro*, preventing the interaction of CUG-MBNL1.	Chen G et al, 2016

			grip strength		
**Ketoprofen**	C2C12 myoblasts	50μM	Upregulated expression of MBNL1 mRNA by 1.2-fold	NSAIDs inhibit the activity of cyclooxygenase enzymes (COX-1 and/or COX-2) and are involved in the synthesis of prostaglandins.<br>The mechanism of action in DM1 of ketoprofen has not been elucidated.	Garcia-Lopez et al, 2008<br>Chen G et al, 2016
**ISOX**	HeLa cells<br><br>Normal and DM1 fibroblasts	EC_{50} = 2.3μM<br><br>5μM for 2 days	Upregulated the expression of *MBNL1* mRNA by 2-fold<br>Partially rescue of the splicing of *SERCA1* e22 and *INSR* e11	Inhibits HDAC6 at low concentrations and HDAC1 and other HDACs at higher concentrations. HDAC inhibition can cause transcriptional arrest. HDAC inhibition appears to have an effect on *MBNL1* mRNA levels.	Zhang et al, 2017
**Vorinostat**	HeLa cells<br><br>Normal and DM1 fibroblasts	EC_{50} = 2.9μM<br><br>5μM for 2 days	Upregulated the expression of *MBNL1* mRNA by 1.8-fold<br>Partially rescue of the splicing of *SERCA1* e22 and *INSR* e11	Inhibits class I and class II HDACs, altering gene transcription and causing cell cycle arrest. HDAC inhibition appears to have an effect on *MBNL1* mRNA levels.	
**INHIBITORS OF H-RAS PATHWAY**					
**Manumycin A**	Clcn1-L minigene reporter assay, C2C12, DM480 plasmid<br><br>HSA^{LR} mice	10-40μM<br><br>3μg	*CLCN1* E7a splicing correction<br><br>Remarkable reduction in *Clcn1* E7A inclusion, reduction of the *Clcn1* mRNA levels, no rescue of *Serca1* E22 and *m-Titin* Mex5	Inhibition of Ras farnesyltransferase, preventing isoprenylation of Ras, required for it to attach to the inner side of the plasma and be activated. H-Ras regulates the splicing of *CLCN1* exon 7A, inhibition of H-Ras prevents inclusion of the exon 7A.<br><br>Reduction of the *Clcn1* mRNA levels suggests another effect on transcription or stability of the *Clcn1* pre-mRNA.	Oana K et al, 2013
**MODULATORS OF PROTEIN KINASES**					
**Ro 31-8220**	DM1 fibroblasts	4.4μM	Foci reduction, release of MBNL1 from foci, splicing rescue of *SERCA1* E22, decrease of steady-state levels of CUGBP1	Inhibition of PKC, preventing the hyperphosphorylation of CUGBP1.<br>Acts through another additional mechanism independently of PKC, suggesting involvement of other kinases.	Kuyumcu-Martinez et al, 2007<br>Wang et al, 2009<br>Ketley A et al, 2014
**Imidazolo-oxindole inhibitorC16**	DM1 myoblasts	1μM	Foci reduction, redistribution of MBNL1, decrease of steady-state levels of CUGBP1, splicing correction of *SERCA1* E22, *DMD* E78, *MBNL*1 E7, *LDB*3 E7, *ITGA*6 E24, *MTMR3* E16 and *SORBS1* E16	*DMPK* mRNA levels remained unchanged, indicating that mutant *DMPK* transcripts are either dispersed within the nucleus without being degraded or liberated to the cytoplasm.<br><br>Inhibits the apoptotic PKR/eIF2a signaling pathway without stimulating the proliferative mTOR/p70s6K signaling mechanism, but it may have selectivity for other kinases.	Wojciechowska M et al, 2014
**Pyrimidine-based inhibitor**	DM1 myoblasts	30μM	Foci reduction, redistribution of MBNL1, decrease of steady-state	C51 inhibits PKR, and it may act through inhibition of hyperphosphorylation of CUGBP1.	Wojciechowska M et al, 2014

**C51**			levels of CUGBP1, splicing correction of *SERCA1* E22, *DMD* E78, *MBNL*1 E7, *LDB*3 E7, *ITGA*6 E24, *MTMR3* E16 and *SORBS1* E16	Inhibition of Src kinases has also been suggested for C51, but its mechanism of action remains undetermined.	
**Metformin**	DM1 MPCs	25mmol/l	Splicing rescue of *INSR* E11, *TNNT2* E5 and *CLCN1* E7a, but the effect was independent of CTG repeats	Activation of AMPK by inhibition of respiratory chain complex I (up to 10mmol/l) and complexes II, IV and V at 25mmol/l.<br><br>Decrease of the tyrosine kinase receptor signaling, which include the epidermal growth factor receptor that controls *INSR* exon 11 inclusion via the inhibition of hnRNPA1 and hnRNPA2B1.<br><br>Activation of additional molecular pathways.	Laustriat et al, 2015
	DM1 myoblasts	25mmol/l	Splicing rescue of *INSR* E11, *TNNT2* E5, *SERCA1* E22, *DMD* E71, *DMD* E78 and *KIF13a* E32		
**AICAR**	DM1 MPCs	2mmol/l	Splicing rescue of *MDM4* E7, *GPCPD1* E5, *CCNL2* E7, *RAGE* E3 and *ZFAND1* E3	Activation of AMPK by inhibition of respiratory chain complex I.	Laustriat et al, 2015
	DM1 myoblasts	2mmol/l	Splicing rescue of *SERCA1* E22, *TNNT2* E5 and *DMD* E71		
**Lithium**	HSA^{LR} mice	20 µM	Reduced levels of GSK3β and normalized levels of Cyclin D3<br>Restored Cugbp1 translational function<br>Improved skeletal muscle strength<br>Reduced myotonia	Lithium competes with Mg^{2+} ions, acting as a co-factor and therefore inhibiting GSK3β. Also, lithium can affect GSK3β indirectly by activating Akt, which increases the phosphorylation/inactivation of the protein.	Jones et al, 2012
**TDZD-8**	HSA^{LR} mice	10mg/Kg for 2 days (i.p. injection)	Reduced levels of GSK3β and normalized levels of Cyclin D3<br>Restored Cugbp1 translational function<br>Improved skeletal muscle strength<br>Reduced myotonia	TDZD-8 is a non-ATP-competitive GSK3β inhibitor that recognizes and interacts with the oxyanion binding site of GSK3β.	
**BIO (6-Bromoindirubin-3'-oxime)**	HSA^{LR} mice	3.6µg/g for 6 weeks every 48 hours (i.p. injection)	Reduced levels of GSK3β and normalized levels of Cyclin D3<br>Restored Cugbp1 translational function<br>Improved skeletal muscle strength<br>Reduced myotonia	Inhibits the phosphorylation on Tyr276/216, a GSK3β activation site, inhibiting this protein. Inhibition of GSK3β restores the translational activity of CUGBP1.	Wei et al, 2017
**SMALL MOLECULES OF NATURAL ORIGIN**					
**Harmine**	DM1 myoblasts	80µM<br>40µM	Foci reduction<br>Increased total levels of MBNL1 and enhanced splicing of *cTNT* E5, *INSR* E11 and *CLCN1* E7a	Harmine binds to RNA and prevents formation of the CUG-MBNL complex.<br><br>Increase of MBNL1 levels by harmine appears to be caused through an additional mechanism.	Herrendorff R et al, 2016
	DM1 fibroblasts	20–80 mM	Splicing rescue of *cTNT* E5 and *INSR* E11		

	HSA^{LR} mice	40 mg/kg	Splicing rescue of *Clcn1* E7a		
